# ﻿*Argyrotaeniasocoromaensis* sp. nov. (Lepidoptera, Tortricidae), a sexually dimorphic micromoth with polyphagous larvae from the arid Andes of northern Chile

**DOI:** 10.3897/zookeys.1189.113678

**Published:** 2024-01-18

**Authors:** Héctor A. Vargas

**Affiliations:** 1 Departamento de Recursos Ambientales, Facultad de Ciencias Agronómicas, Universidad de Tarapacá, Arica, Chile Universidad de Tarapacá Arica Chile

**Keywords:** Andes, arid environments, DNA barcoding, larval polyphagy, new record, new species, sexual dimorphism, taxonomy

## Abstract

*Argyrotaeniasocoromaensis***sp. nov.** (Lepidoptera, Tortricidae, Tortricinae, Archipini) from the arid Andes of northern Chile is described and illustrated. Adults are sexually dimorphic, with differences in wing size, shape and pattern. The larvae feed on *Steviaphilippiana* Hieron. (Asteraceae) and *Lupinusoreophilus* Phil. (Fabaceae). Genetic distance between DNA barcodes of male and female adults reared from larvae collected on the two hosts was 0–0.2% (K2P). The discovery of *A.socoromaensis***sp. nov.** represents the first record of the genus *Argyrotaenia* Stephens, 1852 and the tribe Archipini for the Chilean fauna of Tortricidae.

## ﻿Introduction

The updated, online world catalogue of the family Tortricidae (Gilligan et al. 2018) includes 116 species in the genus *Argyrotaenia* Stephens, 1852 (Tortricinae, Archipini). The widespread Palearctic *A.ljungiana* (Thunberg, 1797), senior synonym of the type species *Tortrixpolitana* Haworth, 1811, is the only Old World representative of the genus, while all the others occur from Canada to Argentina in the New World (Razowski 1997; Brown and Cramer 1999; Gilligan et al. 2018). As part of recent taxonomic studies of the Caribbean fauna of the tribe Archipini, some species included in *Argyrotaenia* by Gilligan et al. (2018) were either transferred to other genera or synonymized, and new ones were described (Austin et al. 2019; Austin and Dombroskie 2020a, b), bringing the current total to 114. Several members of the genus, such as *A.ljungiana* and *A.sphaleropa* (Meyrick, 1909), are pests of cultivated plants (Trematerra and Brown 2004; Gilligan and Epstein 2012; Gonsebatt et al. 2018; Ruiz-Galván et al. 2023).

Forty-three species of *Argyrotaenia* have their type locality in South America (Gilligan et al. 2018). Although some of them were described from Argentina, Bolivia and Peru (e.g., Razowski 1988; Razowski and Becker 2000, 2010; Trematerra and Brown 2004; Razowski and Wojtusiak 2010), the genus remained unknown in neighboring Chile (Razowski and Pelz 2010). However, recent surveys on the arid western slope of the central Andes yielded the first individuals of *Argyrotaenia* in this country. Subsequent morphological examination revealed that they belong to an undescribed species resembling the little-known *A.oriphanes* (Meyrick, 1930), described from Agualani, Puno, Peru. However, the two species can be separated based on differences in wing pattern and genitalia morphology of the male. As the Chilean specimens were reared from larvae collected on plants belonging to two distantly related families and showed differences in size, shape and pattern of male and female forewings, their conspecificity was assessed with mitochondrial DNA sequences of the barcode region (Hebert et al. 2003).

The aim of this contribution is to describe a sexually dimorphic, polyphagous new species of *Argyrotaenia* from the arid western slope of the central Andes, a discovery that represents the first record of this genus and the tribe Archipini from Chile.

## ﻿Material and methods

The adult specimens examined in this study were reared from larvae collected on inflorescences of *Steviaphilippiana* Hieron. (Asteraceae) and *Lupinusoreophilus* Phil. (Fabaceae) in April, 2021 and May, 2023 in the surroundings of Socoroma Village (18°17'22"S, 69°35'12"W) at about 3400 m elevation on the western slope of the Andes in the Parinacota Province of northern Chile. The abdomen of each adult was removed and placed in hot KOH 10% for a few minutes for dissection of the genitalia, which were stained with Eosin Y and Chlorazol Black and mounted on slides with Euparal. The holotype, paratypes and their genitalia slides are deposited in the “Colección Entomológica de la Universidad de Tarapacá” (IDEA), Arica, Chile.

Genomic DNA was extracted from legs of the micromoths using the the QIAamp Fast DNA Tissue Kit (Qiagen). PCR amplification of the barcode region (Hebert et al. 2003) was performed with the primers LCO1490 and HCO2198 (Folmer et al. 1994) using a protocol of 5 min at 94 °C, 35 cycles of 30 s at 94 °C, 30 s at 47 °C, 1 min at 72 °C and a final elongation step of 10 min at 72 °C. DNA purification and sequencing were performed at Macrogen Inc. (Santiago, Chile). The sequences obtained were deposited in the BOLD database of the Barcode of Life Data System (Ratnasingham and Hebert 2007). The software MEGA11 (Tamura et al. 2021) was used to perform sequence alignment with the ClustalW method and to assess the genetic divergence between sequences with the Kimura 2-Parameter (K2P) method.

## ﻿Results

### ﻿DNA barcoding

Three DNA barcode sequences were obtained from the holotype male (BOLD Process ID NCMIC001-23) reared from *S.philippiana*, and the two paratype females (BOLD Process IDs NCMIC002-23, NCMIC003-23) reared from *S.philippiana* and *L.oreophilus*. Genetic divergence between them was 0–0.2% (K2P), confirming that the three specimens belong to a single species with sexually dimorphic adults and polyphagous larvae. The three sequences were clustered under a single Barcode Index Number (BIN) in BOLD (BOLD:AFL1620) with 4.5% p-distance to nearest neighbor.

### ﻿Taxonomy

#### ﻿ Argyrotaenia socoromaensis sp. nov.

Taxon classificationAnimaliaLepidopteraTortricidae

7A9F5387-D71F-5DF2-9931-9DABF40ACCC8

https://zoobank.org/DFA712C0-A92E-427E-8E62-CFB43466FD79

Figs 1 , 2 , 3

##### Type locality.

Chile, Parinacota Province, Socoroma (18°17'22"S, 69°35'12"W), 3400 m elevation on the western slope of the Andes.

##### Type material.

***Holotype***: Chile • ♂; Parinacota, Socoroma; June, 2023; H.A. Vargas leg.; ex-larva inflorescence; *Steviaphilippiana*; May, 2023; “HOLOTYPE *Argyrotaeniasocoromaensis* Vargas” [red handwritten label]; IDEA-LEPI-2023-01; HAV-1661 [genitalia slide]; NCMIC001-23 [BOLD Process ID] (IDEA). ***Paratypes***: CHILE • 1 ♀; same data as for the holotype; IDEA-LEPI-2023-02; HAV-1678 [genitalia slide]; NCMIC002-23 [BOLD Process ID] • 1 ♀; same locality and collector as previous; May, 2021; ex-larva inflorescence; *Lupinusoreophilus*; April, 2021; IDEA-LEPI-2023-03; HAV-1470 [genitalia slide]; NCMIC003-23 [BOLD Process ID] (IDEA).

##### Diagnosis.

Adults of *A.socoromaensis* sp. nov. are sexually dimorphic. The forewing of the holotype male is 10.3 mm long, distal third of the costal margin is almost straight, there are few yellowish-brown scales on the basal fascia, and median fascia is continuous with tornal blotch. In contrast, females have a forewing length of 6.5–8.2 mm, the distal third of the costal margin slightly concave, there are abundant yellowish-brown scales on the basal fascia, and median fascia is conspicuously separated from tornal blotch by the postmedian interfascia. The wing pattern and genitalia of the male of *A.socoromaensis* sp. nov. resemble those of the Peruvian *A.oriphanes* (Clarke 1958, plate 124, figs 4–4b). However, the forewing of *A.socoromaensis* sp. nov. lacks white blotches on the costal half of the basal fascia, and has the median fascia with internal margin strongly sinuous in the middle and external margin slightly sinuous, while the forewing of *A.oriphanes* has broad white blotches on the costal half of the basal fascia, and the medial fascia with internal margin straight in the middle and external margin abruptly indented near the costa. In the male genitalia, the uncus is slightly apically broadened, the sacculus is broadly convex before middle and the phallus is mostly straight in *A.socoromaensis* sp. nov., in contrast with the uncus strongly apically broadened, sacculus with ventral margin straight before middle, and phallus strongly curved of *A.oriphanes*. The female of *A.oriphanes* remains unknown, impeding comparisons.

##### Description.

**Figure 1.**
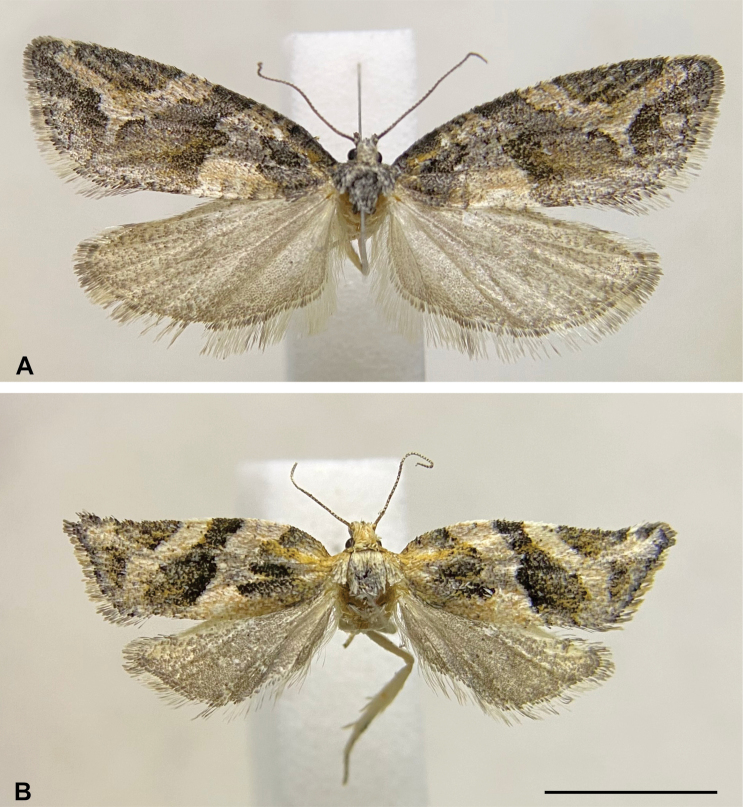
Habitus of *Argyrotaeniasocoromaensis* sp. nov. **A** holotype male, dorsal view **B** paratype female, dorsal view. Scale bar: 5 mm.

**Figure 2.**
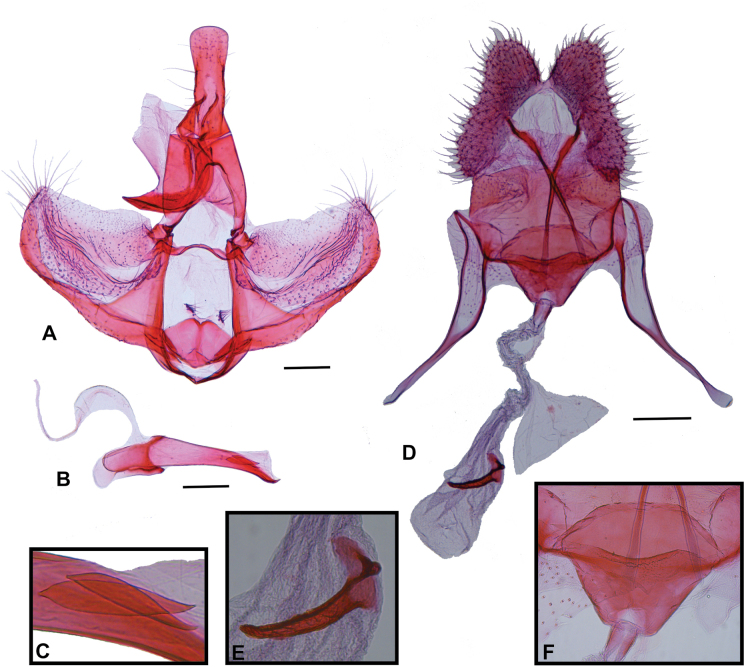
Genitalia of *Argyrotaeniasocoromaensis* sp. nov. **A** male genitalia, ventral view, phallus removed **B** phallus, lateral view **C** cornuti **D** female genitalia, ventral view **E** signum **F** antrum. Scale bars: 0.2 mm.

**Male** (*N* = 1; Fig. 1A). ***Head*.** Vertex mostly whitish gray, dark gray near anterior margin; frons mostly dark gray with a whitish gray transverse stripe near ventral margin. Antenna with scape whitish gray on external surface, dark gray on medial surface, pedicel and flagellum dark gray, flagellum ciliated ventrally. Labial palpus dark gray on external surface, whitish gray on medial surface. ***Thorax*** (forewing length 10.3 mm). Mostly whitish gray and dark gray dorsally with scattered yellowish-brown scales; whitish gray laterally. Foreleg dark gray on external surface, whitish gray on medial surface, tibial epiphysis dark gray; midleg similar to foreleg but whitish gray tibial spurs; hindleg whitish gray with scattered dark gray scales, tibial spurs concolorous. Forewing with distal third of costa almost straight; fasciae and blotches mostly dark gray with scattered whitish gray and yellowish-brown scales; interfasciae mostly whitish gray with scattered yellowish-brown and dark gray scales; fringe gray; basal fascia triangular with external margin trilobed, yellowish-brown scales mostly concentrated near costa; antemedian interfascia with dark gray scales mostly on costal half and yellowish-brown scales mostly on posterior half; median fascia broadening from costa to posterior margin, continuous with tornal blotch, darker near costa, lighter near tornus, internal margin strongly sinuous in the middle, external margin slightly sinuous; postmedian interfascia with narrow, short posterior expansion in the middle not reaching the tornus; subapical blotch semicircular. Hind wing and fringe gray. ***Abdomen*.** Mostly dark gray with scattered whitish gray scales. Male genitalia (Fig. 2A–C). Uncus elongated, anterior third somewhat conical, posterior two-thirds flattened, mostly parallel-sided, slightly broadened apically, apex rounded. Tegumen V-shaped in dorsal view, length in the middle about half of uncus. Gnathos arms as long as uncus, posteriorly curved, distally fused, apex rounded. Vinculum U-shaped. Transtilla a transverse stripe. Juxta diamond-shaped with small V-shaped dorsal excavation with a group of setae near each tip. Valva rectangular, slightly straightening apically, mostly membranous with rounded apical corners; scattered setae, more dense at apex; dorsal margin straight, costa undifferentiated; longitudinal fold lobe-like near transtilla, broadened, slightly sclerotized distally; presaccular gap broad near vinculum, not well-defined toward apex of valva; sacculus narrow, broadly convex before middle, distal third curved to apex of valva. Phallus mostly straight, length similar to valva, progressively narrowing apically, apex slightly curved; caulis small; coecum about a sixth of phallus length, with a keel-shaped antero-ventral projection; vesica with two cornuti.

**Female** (*N* = 2; Fig. 1B). ***Head*.** Vertex and frons yellowish gray. Antenna with scape whitish gray on external surface, dark gray on medial surface, pedicel and flagellum dark gray, flagellum ciliated ventrally. Labial palpus whitish gray on basal half and dark gray with scattered yellowish gray scales on distal half of external surface, whitish gray on medial surface. ***Thorax*** (forewing length 6.5–8.2 mm). Mostly whitish gray dorsally, yellowish gray tegulae; whitish gray laterally. Foreleg mostly dark gray on external surface with scattered yellowish gray scales, whitish gray on medial surface, tibial epiphysis dark gray; midleg mostly whitish gray with scattered dark gray scales, whitish gray tibial spurs; hindleg whitish gray with scattered dark gray scales, tibial spurs whitish gray. Forewing with distal third of costa slightly concave; maculation mainly similar to male, but basal fascia with abundant yellowish-brown scales mostly near tegula; antemedian interfascia with yellowish-brown scales mostly between posterior lobe of the basal fascia and posterior margin of the wing; median fascia with almost uniform width from costa to discal cell, broadened from discal cell to posterior margin of the wing; postmedian interfascia with broad posterior expansion reaching tornus, clearly separating medial fascia from tornal blotch; subapical blotch somewhat triangular. Hind wing similar to male, but termen slightly concave near apex. ***Abdomen*.** Mostly dark gray with scattered whitish gray scales. Female genitalia (Fig. 2D–F). Papillae anales elongate, flattened, narrow, slightly broadened posteriorly, roughened, with setae. Apophyses posteriores about 1.2 times length of papillae anales; apophyses anteriores about 1.4 times length of papillae anales. Sterigma a narrow stripe between the antrum and apophyses anteriores, angled near antrum; antrum somewhat cup-shaped, truncate anteriorly, ventral wall about half the length of dorsal wall, posterior margin of ventral wall slightly convex, posterior margin of dorsal wall rounded; ductus bursae membranous, narrow, about half the length of apophyses anteriores, colliculum about half the length of ventral wall of antrum; ductus seminalis arising near the middle of ductus bursae; corpus bursae membranous, elongated, about 1.5 times the length of ductus bursae, signum spine-like, slightly curved, capitulum small, rounded.

##### Etymology.

The specific epithet is derived from the type locality.

##### Distribution

**Figure 3.**
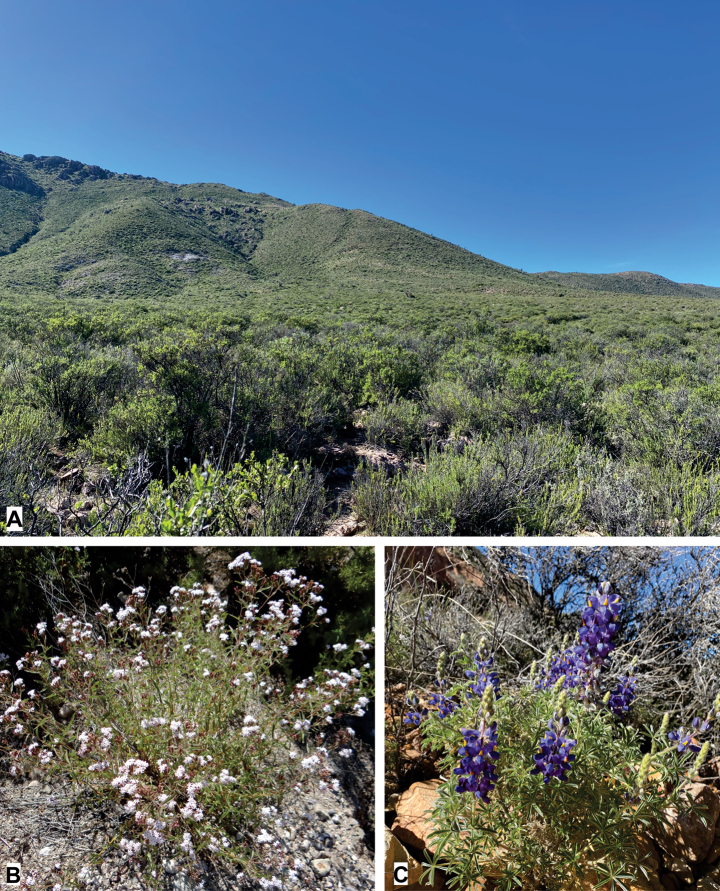
Habitat and host plants of *Argyrotaeniasocoromaensis* sp. nov. **A** habitat at the type locality, near Socoroma Village, at 3400 m elevation on the arid western slope of the Andes of northern Chile **B***Steviaphilippiana* Hieron. (Asteraceae) **C***Lupinusoreophilus* Phil. (Fabaceae).

**(Fig. 3A).***Argyrotaeniasocoromaensis* sp. nov. is known only from the type locality in the surroundings of Socoroma Village, at about 3400 m elevation on the western slope of the Andes in the Parinacota Province, northern Chile.

##### Host plants

**(Fig. 3B, C).** The adult specimens of *A.socoromaensis* sp. nov. examined in this study were reared from larvae collected on inflorescences of *Steviaphilippiana* Hieron. (Asteraceae) and fruits of *Lupinusoreophilus* Phil. (Fabaceae).

## ﻿Discussion

The uniqueness of the fauna of Tortricidae of Chile has been widely recognized (Obraztsov 1964; Razowski 1988; Brown 1999; Razowski and Pelz 2010). More than 80% of the species recorded in this country are endemic (Urra 2020). South-central Chile also harbors some endemic genera, among them *Accuminulia* Brown, 1999, *Parvulia* Urra, 2016 and *Natria* Urra, 2020 (Brown 1999; Urra 2016, 2020), while some other genera inhabiting this area have relatively narrow geographic ranges in South America with additional records restricted to neighboring countries, as in the case of *Chileulia* Powell, 1986, *Proeulia* Obraztsov, 1964 and *Ptychocroca* Brown & Razowski, 2003 (Brown and Razowski 2003; Razowski and Pelz 2010; Cepeda and González 2015). In contrast, species recorded in northern Chile belong to more widespread genera, such as *Cryptophlebia* Walsingham, 1900 and *Strepsicrates* Meyrick, 1888 (Clarke 1987; Vargas-Ortiz and Vargas 2018). The discovery of *A.socoromaensis* sp. nov. reinforces this pattern by adding the record of another widespread genus to the arid environments of northern Chile.

Host plant records of the Palearctic *A.ljungiana* and many Nearctic *Argyrotaenia* suggest that polyphagy is very common in this genus, while a few species have narrower host ranges, feeding on plants belonging to a single family (Brown et al. 2008). Such variation in host ranges can occur even among members of the same species group (Landry et al. 1999). Host plants have been recorded for only a few Neotropical species (Brown et al. 2008). Among the South American fauna, published host records mainly involve *A.loxonephes* (Meyrick, 1937) and *A.sphaleropa* (Meyrick, 1909), two remarkably polyphagous pest species whose larvae feed on plants belonging to 16 and 21 families, respectively (Trematerra and Brown 2004). The polyphagy of *A.socoromaensis* sp. nov. fits the more common host range currently recognized for the genus.

Sexual dimorphism, mostly related to wing pattern, has been documented for several members of *Argyrotaenia* (Obraztsov 1961; Powell 1965; Austin and Dombroskie 2020a). Correct association of males and females can be particularly difficult in species with marked sexual dimorphism, as in the case of *A.montezumae* (Walsingham, 1914), whose female was originally described under another specific name currently recognized as a synonym (Obraztsov 1961; Gilligan et al. 2018). The remarkable sexual dimorphism of *A.socoromaensis* sp. nov. involves differences in wing size, shape, and pattern.

DNA barcodes have been used successfully to explore host plant ranges and to associate females and males of sexually dimorphic species of Tortricidae (Hulcr et al. 2007; Corley and Ferreira 2017; Austin and Dombroskie 2020a). Although the description provided here for *A.socoromaensis* sp. nov. is based on only three specimens, the analysis of their DNA barcodes accurately supports the recognition of the male holotype and the two female paratypes as members of a single species with sexually dimorphic adults and polyphagous larvae. The discovery of *A.socoromaensis* sp. nov. raises to 105 the species and 38 the genera recorded for the Chilean fauna of Tortricidae (Razowski and Pelz 2010; Cepeda and Curkovic 2020; Urra 2020), and highlights the need to explore further the overlooked diversity of micromoths of the natural environments of the arid western slopes of the central Andes.

## Supplementary Material

XML Treatment for Argyrotaenia socoromaensis

## ﻿Additional information

### Author ORCIDs

Héctor A. Vargas https://orcid.org/0000-0002-5355-3157

### Data availability

All of the data that support the findings of this study are available in the main text.
